# Proteomics, a systems biology based approach to investigations of *Jatropha curcas* seeds

**DOI:** 10.1186/1753-6561-5-S7-P162

**Published:** 2011-09-13

**Authors:** Fatemeh Maghuly, Stefan Kogler, Gorji Marzban, Katharina Nöbauer, Ebrahim Razzazi, Margit Laimer

**Affiliations:** 1 Plant Biotechnology Unit (PBU), Dept. Biotechnology, University of Natural Resources and Life Sciences, Muthgasse 18, 1190 Vienna, Austria; 2 VetCore Facility of Technology, Proteomics Unit, University of Veterinary Medicine, 1210 Vienna, Austria

## Background

*Jatropha curcas*(*Euphorbiaceae*) is cultivated for harvesting the unique oil contained in its seeds, which can be used as raw material for the production of biodiesel [1]. The seed cake remaining after pressing can not be used for animal feed, because it contains toxic proteins and other compounds, e.g. curcin, phorbol esters. The development of genotypes better suited for the production of biodiesel showing lower levels of toxic/allergenic proteins is being hampered by a lack of understanding the a) metabolic pathways and enzymes leading to the production of fatty acids during seed development and b) role of proteins deposited during seed development. In recent years the field of proteomic research has become a fast growing discipline with high relevance to biological sciences.

While genomic and transcript-profiling studies have provided a wealth of information about different plant developmental processes, there is growing awareness that the abundance of mRNA transcripts is not always representative of protein levels and that mechanisms of post-translational regulation must also play an important role.

Proteomics is a systems biology based approach investigating the whole expressed proteins at a given time point and under certain condition [2]. It has been shown to be a valuable tool for studying the biology of living organisms and their interaction with the environment in post genomics area. Proteomics has been shown to have potential values to deliver knowledge about complex biochemical processes and is being used in various fields of modern botany and agriculture like plant biomarker discovery related to resistance as well as drought and water stress. Furthermore proteomics offer major advantages linked to its high throughput capacity and its ability to perform simultaneously the analysis of hundreds of proteins from the same samples.

## Methods

The method of choice for proteome analysis is the combination of high resolution protein separation like two dimensional gel electrophoresis (2-DE) with tandem mass spectrometric (MS/MS) identification of proteins. The two dimensional gel electrophoresis separates proteins based on their isoelectric points (isoelectric focusing) and in a second dimension based on their molecular weight [3].

In this study a proteomic approach was conducted in order to identify the expression patterns of interesting proteins during seed development, as well as toxic and/or allergenic proteins in Jatropha, 2-DE coupled with mass spectrometry and *de novo* sequencing, were employedto analyze whole seed proteins of six developmental stages, covering the essential ontological phases of these important plant organs.

Prominent spots identified in 2-DE analyses were excised, washed, reduced with DTT and alkylated with iodoacetamide. The in-gel digest with trypsin was carried out for 8 hours. The peptides were de-salted using µZip-Tips C18 (Millipore) and 0.5 µl were spotted onto a disposable AnchorChip MALDI target plate pre-spotted with a-cyano-4-hydroxycinnamic acid (PAC target, Bruker Daltonics). Data were acquired on a Matrix Assisted Laser Desorption Ionisation Tandem Time-of-Flight (MALDI-TOF/TOF) mass spectrometer (Ultraflex II, Bruker Daltonics) in MS and MS/MS modes. Spectra processing and peak annotation were carried out using FlexAnalysis and Biotools (Bruker Daltonics).

In order to enhance the quality of tandem mass spectrometry (MS/MS) spectra for de novo sequencing, N-terminal chemical modification using 4-sulfophenyl isothiocyanate (SPITC) was carried out for 1 h at 56°C [4]. The derivatised peptides were de-salted a second time. Again 0.5 µL were spotted onto a PAC target and analyzed as described above.

For standard database searches, processed spectra were searched via Mascot2 in the Swiss-Prot database and in NCBInr. Identifications were considered statistically significant where p < 0.05. Peptide deNovo-sequencing was carried out manually using FlexAnalysis. The sequences were then used for a homology search using MS-homology with the following search parameters: taxonomy Viridiplantae using pre-search parameter 9VIRI (organism code from the UniProt Knowledgebase); Database UniProtKB.2011.01.11; Score Matrix BLOSUM62; 30% amino acid exchanges allowed.

## Results and conclusions

In 2-DE analyses it was possible to identify 100 non-redundant protein spots. Identified proteins belonged to different functional classes, including proteins involved in metabolism, protein destination and storage, and energy metabolism, which were highly representative.

The significant change in abundance of protein spots during the different developmental stages of seeds indicated that several pathways were involved in the biosynthesis of these interesting compounds, but vary during the growth phases of plant organs, in this case seeds.
